# Developing a multi-level human-centered design framework for digital health implementation: a cross-project methodological synthesis

**DOI:** 10.3389/fdgth.2026.1939519

**Published:** 2026-10-28

**Authors:** Md Shafiqur Rahman Jabin, Ronald Danny Nyatuka, Paul Macharia

**Affiliations:** 1 Department of Medicine and Optometry, Linnaeus University, Kalmar, Sweden; 2 School of Computing and Engineering Sciences (SCES), Strathmore University, Nairobi, Kenya

**Keywords:** digital health implementation, health systems strengthening, implementation research, implementation science, lifecycle methodology, low-resource settings, participatory design, socio-technical systems

## Abstract

**Background:**

Digital health interventions have considerable potential to improve healthcare access, quality, and efficiency. Despite increasing adoption, many interventions fail to achieve sustainable implementation or progress beyond pilot phases because they are insufficiently integrated with user needs, organizational workflows, and health system contexts. Although human-centered design (HCD) has improved the usability and acceptability of digital health technologies, its application frequently remains confined to early design activities and is rarely integrated with implementation science throughout the implementation lifecycle.

**Objective:**

This study aimed to develop a multi-level, human-centered design framework informed by a cross-project methodological synthesis integrating participatory design and implementation science to support the design, implementation, evaluation, and scale-up of digital health interventions.

**Methods:**

A cross-project methodological synthesis was conducted using complementary digital health implementation studies undertaken in Kenya. The empirical corpus included studies spanning adolescent mental health, neonatal clinical decision support, digital health infrastructure, generative artificial intelligence, and frugal digital health innovation. Framework development followed an iterative analytical process combining thematic synthesis, constant comparative analysis, and abductive reasoning. The synthesis organized recurring methodological themes into an integrated framework spanning user, workflow, and system perspectives across the digital health implementation lifecycle.

**Results:**

The synthesis identified recurring methodological processes that consistently contributed to successful digital health implementation, including contextual understanding, participatory stakeholder engagement, iterative refinement, workflow integration, organizational readiness, governance, and sustainability. These findings informed the development of a multi-level framework structured around six iterative implementation phases: Understand, Co-design, Prototype, Evaluate, Implement, and Scale, and three interconnected analytical levels: user (micro), workflow (meso), and system (macro). The framework conceptualizes digital health implementation as a continuous sociotechnical process in which sustained adoption and scale-up depend upon coordinated alignment among these three levels throughout the implementation lifecycle.

**Conclusions:**

This study advances human-centered design by integrating it with implementation science through an empirically derived, lifecycle-oriented methodological framework for digital health implementation. The proposed framework may provide researchers, developers, practitioners, and policymakers with a structured approach for designing, implementing, evaluating, and scaling digital health interventions while supporting sustainable, context-responsive, and equitable digital health transformation.

## Introduction

Digital health technologies are increasingly positioned as critical tools for strengthening health systems, improving access to care, and addressing global health inequities (1, 2). Innovations such as mobile health (mHealth), artificial intelligence (AI), and digital platforms have demonstrated potential to enhance service delivery, particularly in low- and middle-income countries (LMICs), where resource constraints and workforce shortages are prominent (3, 4). However, despite substantial investment and rapid technological advancement, many digital health interventions fail to progress beyond pilot stages or achieve sustainable integration into routine healthcare systems (5, 6).

This persistent “pilot-to-scale gap” has been widely documented in digital health research and is often attributed to a mismatch between technological solutions and the complex social, organizational, and systemic environments in which they are implemented (7). Interventions are frequently designed with a strong emphasis on technical functionality, while insufficient attention is given to user needs, workflow integration, and health system readiness. As a result, even well-designed tools may face barriers such as low adoption, poor usability, workflow disruption, and limited scalability (8, 9).

Human-centered design (HCD) has emerged as a promising approach to address these challenges. HCD emphasizes iterative, participatory processes that actively involve end users and stakeholders throughout the design and development lifecycle, ensuring that interventions are aligned with real-world needs and contexts (10, 11). In digital health, HCD has been associated with improved usability, acceptability, and user engagement, particularly in interventions targeting adolescents and underserved populations (12, 13).

Despite its growing adoption, HCD in digital health remains fragmented. Many studies focus on discrete phases of the design process, such as co-design or usability testing, rather than applying HCD across the full lifecycle of intervention development, implementation, and scale-up (14). Furthermore, there is limited integration between HCD and implementation science frameworks, which emphasize the importance of contextual and system-level determinants, including workflow alignment, governance structures, infrastructure capacity, and organizational readiness, in shaping implementation outcomes (6, 9, 14–16). Although several human-centered design frameworks and implementation science models have been proposed, they generally address different phases of digital health innovation rather than offering an integrated methodological approach across the full implementation lifecycle. Human-centered design frameworks predominantly emphasize understanding users, participatory co-design, and usability optimization, whereas implementation science frameworks such as the Consolidated Framework for Implementation Research (CFIR) and the Nonadoption, Abandonment, Scale-up, Spread, and Sustainability (NASSS) framework primarily focus on contextual determinants influencing adoption, implementation, and sustainability (6, 8, 17, 18). Consequently, researchers and practitioners often lack practical guidance on how to systematically integrate user-centered design activities with workflow optimization, organizational readiness, governance, and scale-up throughout digital health implementation.

Emerging evidence suggests that successful digital health interventions require alignment across multiple levels, including individual users, operational workflows, and broader health systems (19, 20). However, existing HCD frameworks rarely incorporate these multi-level dynamics, limiting their ability to guide real-world implementation and scale-up in complex, resource-constrained settings. Recent methodological guidance also emphasizes that evaluating digital health interventions requires approaches that go beyond usability assessment to include implementation processes, contextual adaptation, stakeholder engagement, organizational integration, and long-term sustainability (4, 5). Nevertheless, methodological guidance remains limited on how to integrate these complementary activities into a coherent human-centered design process that supports implementation from early contextual exploration through large-scale deployment. Addressing this methodological gap is increasingly recognized as a priority for advancing digital health evaluation and implementation research.

To address these methodological and implementation gaps, this study develops a multi-level human-centered design framework for digital health implementation through a cross-project methodological synthesis of multiple digital health implementation studies conducted in Kenya. Unlike existing frameworks that primarily emphasize either participatory design or implementation determinants, the proposed framework integrates evidence across diverse digital health interventions representing community-based, clinical, and health system contexts. By synthesizing empirical evidence from qualitative inquiry, co-design, usability evaluation, implementation research, and system-level analyses, this study extends human-centered design beyond user-focused approaches to encompass workflow integration, organizational readiness, governance, and scalability. The resulting framework provides a structured methodological approach to designing, evaluating, implementing, and scaling digital health interventions throughout the full implementation lifecycle, with particular relevance for resource-constrained health systems. To situate the proposed framework within the existing literature and demonstrate its methodological contribution, Table 1 compares the scope and key characteristics of commonly used human-centered design and implementation science frameworks with those of the proposed multi-level human-centered design framework.

**Table 1:** Comparison of existing human-centered design and implementation science frameworks with the proposed multi-level human-centered design framework for digital health implementation.

Framework	User-centered design	Workflow integration	System readiness	Full implementation lifecycle	Scalability	Empirical synthesis
Human-centered design (HCD) (10)	✓	Limited	Limited	Partial	Limited	No
User-centered design (UCD) (21)	✓	Limited	No	Early stages	No	No
Consolidated Framework for Implementation Research (CFIR) (8, 17)	Partial	✓	✓	Implementation	✓	No
Nonadoption, Abandonment, Scale-up, Spread, and Sustainability (NASSS) (6, 18)	Partial	✓	✓	Full	✓	No
Systems Engineering Initiative for Patient Safety (SEIPS 3.0) (22)	✓	✓	Partial	Implementation	Partial	No
Proposed multi-level HCD framework	✓	✓	✓	✓	✓	✓

Collectively, these gaps highlight the need for a more comprehensive methodological approach that integrates participatory design, workflow integration, and system-level implementation considerations throughout the digital health implementation lifecycle. The following section reviews the conceptual foundations underpinning this integrated perspective.

## Conceptual background

### Human-centered design in digital health

Human-centered design (HCD) is a multidisciplinary approach that emphasizes designing technologies and interventions around end users' needs, preferences, and lived experiences. Rooted in design science and human–computer interaction, HCD involves iterative cycles of problem exploration, ideation, prototyping, and evaluation, with active stakeholder engagement throughout the process (23, 24). In digital health, HCD has gained increasing attention as a means of improving usability, acceptability, and user engagement, particularly in interventions targeting complex and sensitive domains such as mental health and adolescent health (10, 12).

Closely related to HCD, user-centered design (UCD) has historically focused on optimizing usability and user-system interaction. While UCD emphasizes usability and interface design, HCD extends beyond this to incorporate broader contextual, cultural, and social dimensions of user experience (25, 26). In practice, researchers often use the terms interchangeably in digital health research, though HCD is increasingly preferred for its broader, systems-oriented perspective.

Beyond improving usability, HCD has increasingly been recognized as a methodological approach for addressing complex sociotechnical challenges in healthcare. Rather than viewing digital technologies as isolated technical solutions, HCD acknowledges that successful implementation depends on continuous interaction among users, technologies, organizational processes, and contextual environments. Consequently, contemporary HCD extends beyond interface optimization to encompass stakeholder engagement, contextual inquiry, iterative learning, and adaptation throughout the digital health lifecycle (10, 27–30). However, practical guidance on operationalizing these broader principles across implementation and scale-up remains limited, particularly in LMICs.

HCD applications in digital health have improved intervention relevance, engagement, and equity. Participatory approaches, including co-design and co-creation, enable stakeholders—such as patients, community members, and healthcare providers- to actively shape intervention design, ensuring alignment with real-world needs (13, 31). This is particularly important in LMIC settings, where contextual variability, resource constraints, and cultural factors significantly influence technology adoption and use (32).

However, despite its strengths, HCD is often applied in a fragmented manner. Many studies focus primarily on early design phases, such as needs assessment or co-design workshops, with less attention to later implementation and scale-up stages. As a result, even well-designed interventions may still face barriers related to workflow integration, organizational readiness, and system-level constraints.

### Implementation science and digital health

Implementation science offers a complementary perspective by focusing on the processes and contextual factors that shape intervention adoption, integration, and sustainability in real-world settings. Frameworks such as the Consolidated Framework for Implementation Research (CFIR) and the Nonadoption, Abandonment, Scale-up, Spread, and Sustainability (NASSS) framework highlight the importance of multi-level determinants, including individual, organizational, and system-level factors (6, 8).

These frameworks emphasize that successful implementation depends not only on an intervention's intrinsic quality but also on its compatibility with existing workflows, organizational structures, and broader policy environments. For example, CFIR identifies domains such as inner setting (organizational culture, resources), outer setting (policy and funding), and intervention characteristics as key determinants of implementation outcomes (8). Similarly, the NASSS framework highlights the complexity of digital health interventions and the challenges associated with scaling innovations within dynamic health systems (6, 33).

Although implementation science frameworks provide valuable insights into the contextual determinants of adoption and sustainability, they generally offer limited guidance on engaging end users throughout intervention design and refinement. Consequently, implementation planning often begins after key design decisions have already been made, potentially limiting opportunities to address usability, contextual adaptation, and stakeholder priorities during technology development. Recent work has explicitly examined how implementation science can be integrated into human-centered design for digital health. For example, Waddell et al. (64) integrated the Nonadoption, Abandonment, Scale-up, Spread, and Sustainability (NASSS) framework into the Double Diamond design process, showing how implementation considerations can be incorporated into design, iteration, and refinement rather than addressed only after intervention development. Integrating implementation science with HCD therefore offers an opportunity to establish a continuous methodological pathway linking participatory design, implementation readiness, organizational integration, and long-term sustainability (9, 34).

Importantly, integrating HCD and implementation science goes beyond adding implementation determinants to an otherwise design-focused process. Waddell et al. (64) show how implementation science can be incorporated into the design process itself, providing a practical bridge between HCD activities and implementation considerations. Building on this contribution, the present framework extends integration across three interconnected socio-technical levels, i.e., user (micro), workflow (meso), and system (macro), and across six iterative implementation phases: Understand, Co-design, Prototype, Evaluate, Implement, and Scale. The framework therefore operationalizes HCD and implementation science as a continuous, multi-level methodological process rather than as sequential or separate activities.

In digital health, implementation science has been instrumental in explaining why many interventions fail to scale despite promising pilot results. Common barriers to adoption include workflow disruption, increased cognitive burden, inadequate training, and misaligned governance (35, 36). These insights underscore the importance of considering not only user experience but also operational and structural contexts when designing and implementing digital health interventions.

### Toward a multi-level perspective

While HCD and implementation science offer complementary strengths, researchers often apply them in isolation. HCD prioritizes user experience and participatory design, whereas implementation science focuses on system-level determinants and real-world integration. This separation limits existing approaches from fully addressing the complexity of digital health implementation.

Emerging literature suggests the need for integrated approaches that bridge design and implementation perspectives. Digital health interventions operate within multi-level socio-technical systems, where outcomes are shaped by interactions between individuals, workflows, and broader organizational and policy environments (6, 20, 37, 38). Across the digital health lifecycle, these interacting levels should not be viewed as independent domains but rather as mutually reinforcing components of a complex adaptive system in which design decisions, implementation processes, and organizational contexts continuously influence one another. Table 2 presents a conceptual synthesis, not a direct output of the cross-project empirical analysis, synthesizing the three interacting levels identified in the HCD and implementation science literature.

**Table 2:** Multi-level dimensions underpinning the proposed human-centered digital health implementation framework.

Level	Primary focus	Representative components	Expected implementation outcome
User (Micro)	Individual needs and experiences	Usability, accessibility, engagement, acceptability, trust	Improved adoption and sustained user engagement
Workflow (Meso)	Integration into routine practice	Task alignment, cognitive workload, interoperability, clinical workflow	Efficient implementation and reduced workflow disruption
System (Macro)	Organizational and health system readiness	Governance, infrastructure, workforce capacity, financing, policy, sustainability	Long-term implementation, institutionalization, and scale-up

The conceptual synthesis presented in Table 2 demonstrates that human-centered design and implementation science offer complementary strengths but remain insufficiently integrated in existing approaches. Building on these theoretical foundations, the Methods section describes the cross-project methodological synthesis undertaken to develop the proposed integrated framework.

Although these multi-level dynamics are recognized, existing HCD frameworks rarely incorporate them explicitly. Similarly, implementation science frameworks often lack detailed guidance on participatory design processes. This gap highlights the need for a unified conceptual approach that integrates human-centered design with implementation science.

### Conceptual gap and study contribution

In summary, while HCD has improved the design of digital health interventions and implementation science has advanced understanding of real-world adoption, frameworks that integrate these perspectives across the full lifecycle of digital health development and deployment remain lacking. Existing approaches tend to focus on either design or implementation, rather than the continuous interaction among user experience, workflow integration, and system readiness.

To address these limitations, this study develops a multi-level human-centered design framework through a cross-project methodological synthesis of complementary digital health implementation studies. Unlike existing frameworks that primarily emphasize either participatory design or implementation determinants, the proposed framework integrates evidence across multiple implementation contexts to establish a continuous methodological pathway spanning contextual understanding, participatory co-design, iterative development, usability evaluation, implementation readiness, governance, and scale-up. By explicitly linking user, workflow, and system levels throughout the digital health implementation lifecycle, the framework offers practical methodological guidance for researchers, implementers, policymakers, and digital health developers seeking to design, evaluate, implement, and sustain digital health interventions in complex healthcare environments. To illustrate the framework's theoretical foundation, Figure 1 presents the conceptual relationships among human-centered design, implementation science, and digital health implementation, highlighting the rationale for integrating these complementary perspectives.

**Figure 1:**
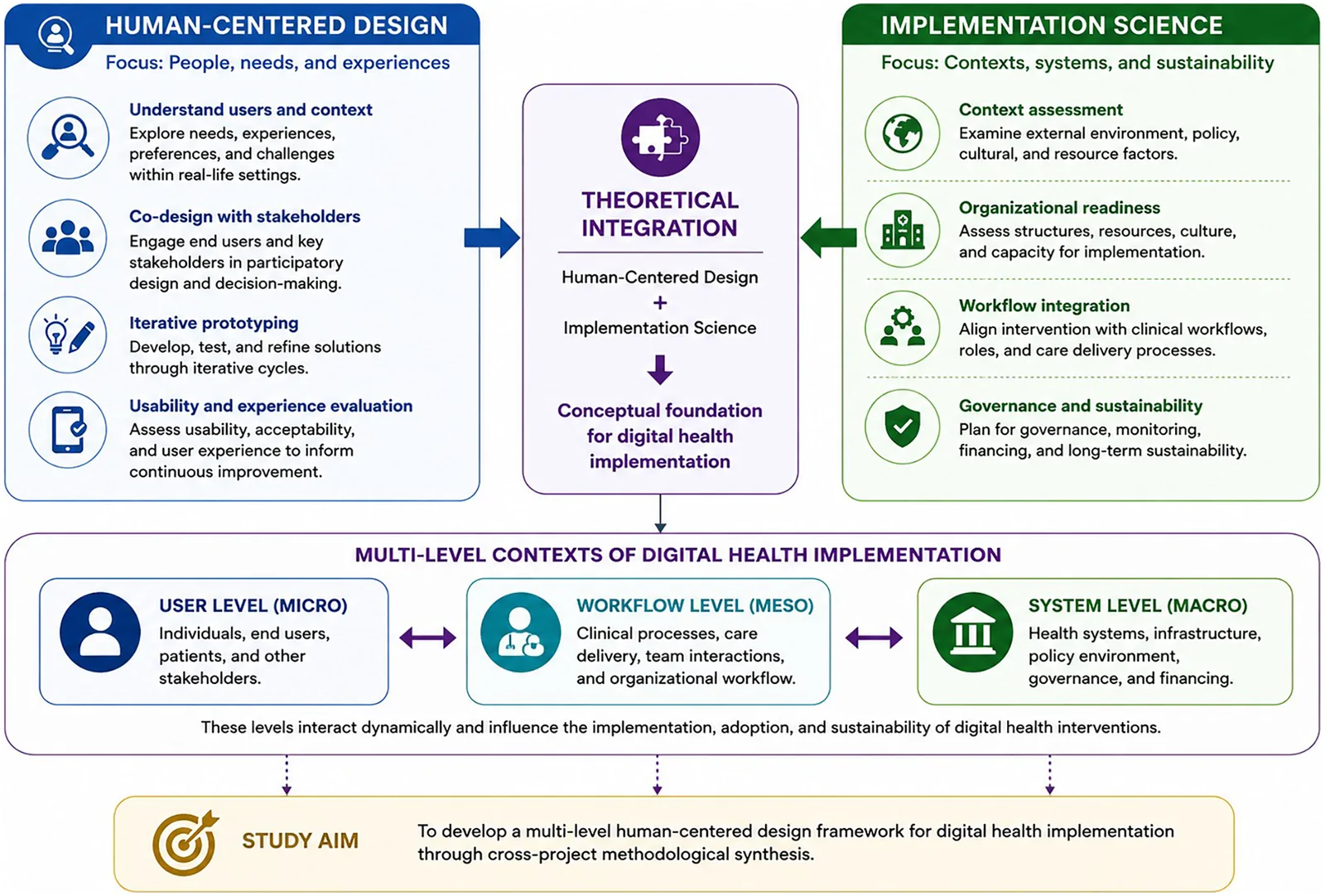
Conceptual relationship between human-centered design, implementation science, and digital health implementation.

Figure 1 illustrates the complementary roles of human-centered design and implementation science in digital health. It demonstrates how integrating user-centered design principles with implementation determinants provides the conceptual foundation for a multi-level approach to digital health implementation that considers users, workflows, and health systems throughout the implementation lifecycle.

## Methods

### Study design and approach

This study employed a cross-project qualitative synthesis to develop a multi-level human-centered design (HCD) framework for digital health implementation. The synthesis comprised five completed digital health research projects conducted by the same research team in Kenya between 2022 and 2026. This shared research context was both a strength, because it enabled consistent methodological comparison across projects, and a potential source of analytic bias, because the team's established use of human-centered and participatory approaches may have influenced both the studies included and the methodological patterns identified. The approach drew on established methods for qualitative synthesis and theory-building, aiming to generate a conceptual framework grounded in empirical evidence rather than purely theoretical abstraction (39).

The methodological synthesis drew on a purposively selected corpus of five completed digital health projects the research team conducted in Kenya between 2022 and 2026. Projects were eligible for inclusion when they (1) had completed substantive empirical activities, (2) addressed digital health development, implementation, or scale-up, (3) incorporated human-centered, user-centered, participatory, implementation science, or design science approaches, and (4) generated methodological evidence relevant to at least one stage or level of digital health implementation (39, 40). Projects were selected to provide complementary methodological experiences across different health priorities, stakeholder groups, technologies, implementation settings, and lifecycle stages rather than to represent a statistically representative sample. This purposive approach enabled comparison of recurring methodological processes while preserving each project's contextual characteristics.

Unlike conventional systematic reviews or meta-syntheses that aggregate evidence across unrelated studies, the present methodological synthesis focused on a coherent program of interconnected digital health research. The included projects shared common methodological foundations rooted in human-centered design, user-centered design, implementation science, participatory research, or design science, while addressing distinct implementation contexts including adolescent mental health, neonatal care, digital health infrastructure, and generative artificial intelligence. This enabled analytical comparison of recurring methodological patterns while maintaining consistency in research philosophy, implementation context, and overall study objectives. To enhance transparency about the empirical basis of the methodological synthesis, Table 3 summarizes the characteristics, scale, implementation settings, and methodological contributions of the digital health implementation studies that informed the framework's development.

**Table 3:** Characteristics of the digital health implementation studies included in the cross-project methodological synthesis.

Study	Digital health domain	Project status/empirical evidence synthesized	Design approach	Primary participants	Scale and implementation context	Main data sources	Principal methodological contribution
AI Mental Health Chatbot (41)	Adolescent mental health	Completed research program with ongoing dissemination of empirical outputs; synthesis informed by completed empirical phases, including contextual inquiry, participatory co-design, usability evaluation, and implementation findings	Human-centered design; participatory co-design	Adolescents and young adults	Implemented across contrasting urban, peri-urban, and rural settings in Kenya, providing variation in community and implementation context.	Focus group discussions, co-design workshops, usability testing, surveys	Contextual understanding, participatory co-design, iterative refinement
Neonatal Risk Predictor (21, 42)	Clinical decision support	Completed empirical implementation study with ongoing dissemination of empirical output, informing workflow evaluation and implementation readiness	User-centered design; implementation science	Healthcare professionals	Implemented across three health facilities and included approximately 394 newborn assessments over four months, providing a facility-based implementation context.	Key informant interviews, workflow mapping, usability evaluation, implementation assessment	Workflow integration, implementation readiness, and feasibility evaluation
Bright Health (43, 44)	Digital health infrastructure	Completed empirical implementation study	Human-centered implementation; stakeholder engagement	Health system stakeholders	Implemented across 12 public healthcare facilities in rural and peri-urban Kenya, providing geographically and operationally diverse implementation contexts.	Interviews, stakeholder workshops, implementation planning	Infrastructure readiness, governance, sustainability
Generative AI for Healthcare (45)	Clinical generative artificial intelligence	Completed empirical implementation study with ongoing dissemination of empirical outputs	User-centered design; design science	Healthcare professionals	Implemented across multiple Kenyan counties and health-system contexts, involving healthcare professionals and wider health-system stakeholders.	Baseline surveys, usability evaluation, iterative refinement	AI customization, usability engineering, implementation evaluation
Frugal Innovation Research Program (37, 38)	Maternal health and health information systems	Program of completed empirical studies synthesized across multiple implementation activities	Frugal innovation; participatory approaches	Health professionals and policymakers	Included rural and urban-poor implementation contexts in Kenya, enabling comparison across contrasting resource and service-delivery environments.	Interviews, surveys, contextual analysis, framework development	Contextual adaptation, implementation barriers, sustainable digital innovation

The methodological diversity represented across the included studies provided a robust empirical foundation for identifying recurring implementation processes and methodological patterns. The analytical procedures used to synthesize these findings and develop the proposed framework are described in the following section.

### Data sources and cross-project evidence base

The methodological synthesis drew on a purposively selected corpus of complementary digital health implementation studies the research team conducted in Kenya between 2022 and 2026. The empirical corpus comprised completed studies representing adolescent mental health, neonatal clinical decision support, digital health infrastructure, generative artificial intelligence, and frugal digital health innovation. Although these studies addressed different health priorities and stakeholder groups, they shared common methodological foundations, including human-centered design (HCD), user-centered design (UCD), participatory research, implementation science, and design science approaches, thereby enabling meaningful cross-project methodological synthesis (39, 46).

Rather than aggregating intervention outcomes, the synthesis focused on identifying methodological processes and implementation experiences that could inform framework development. The synthesis examined findings from interviews, focus groups, co-design activities, and other empirical sources for evidence of how researchers and stakeholders explored context, identified needs, generated and refined requirements, evaluated usability and workflow fit, addressed implementation barriers, and adapted interventions. Thus, these data served as evidence of methodological processes rather than being re-analyzed to generate new substantive findings about participants or intervention outcomes (46).

Data sources included qualitative interviews, focus group discussions, participatory co-design workshops, usability and user experience evaluations, implementation assessments, workflow observations, stakeholder consultations, project reports, and implementation documentation. These sources were not re-analyzed to estimate intervention effectiveness; instead, we examined previously generated and analyzed project findings for evidence on how methodological activities were conducted, how stakeholder perspectives informed design decisions, how workflow and implementation issues were identified, and how contextual or system-level factors shaped implementation. Methodological and project documentation, including study protocols, methodological write-ups, project reports, and implementation documentation where available, was used to contextualize how empirical evidence had been generated and interpreted within each project (47).

### Analytical framework and cross-project synthesis

Framework development drew on thematic synthesis and abductive analytical reasoning, with analysis proceeding iteratively between project-level empirical findings, methodological interpretations, and relevant theoretical concepts (39, 48–50). First, previously analyzed findings from each project were examined independently to identify methodological processes, stakeholder engagement practices, implementation experiences, workflow considerations, and contextual influences. These observations were organized into descriptive methodological codes while retaining their originating project and context. Second, codes and preliminary themes were compared across projects using constant comparison to identify recurring processes, similarities, contextual differences, and relationships. Third, related concepts were synthesized into higher-order themes that represented methodological patterns extending across more than one project.

The abductive component involved iterative movement between empirical observations and existing HCD and implementation science concepts. When a recurring observation was identified, we considered whether existing concepts adequately explained it; where they did not, the interpretation was refined through further comparison with the empirical corpus and relevant theoretical literature. Deductive interpretation was used as a sensitizing and organizing process rather than as a fixed coding framework. HCD concepts were considered alongside implementation science perspectives to examine how design activities interacted with user, workflow, organizational, and system-level implementation conditions.

Constant comparison was conducted both within and across projects. Within projects, methodological activities and implementation experiences were compared across stages of the digital health lifecycle; across projects, recurring and divergent patterns were examined in relation to differences in health domain, stakeholder group, technology, and implementation context. This iterative comparison generated higher-order themes, which were then mapped onto the three analytical levels (micro/user, meso/workflow, and macro/system) and the six implementation lifecycle phases to construct and refine the proposed framework.

The synthesis adopted a constant comparative analytical strategy to identify recurring methodological characteristics across the included studies. Rather than comparing intervention effectiveness, the analysis focused on identifying common methodological processes, stakeholder engagement strategies, implementation determinants, workflow characteristics, and contextual factors that consistently influenced digital health implementation. This iterative comparison enabled the development of higher-order analytical themes that subsequently informed the construction of the proposed framework (51).

### Framework development process

Framework development followed an iterative, cross-project analytical process with three interconnected phases: within-study analysis, cross-project methodological synthesis, and framework construction and refinement. Throughout all phases, analytical decisions were guided by continuous comparison between empirical findings and relevant human-centered design and implementation science concepts, enabling iterative refinement of analytical interpretations while staying closely aligned with the original data (39, 48).

The analytical strategy combined inductive identification of methodological patterns from previously analyzed project findings with deductive interpretation informed by relevant human-centered design and implementation science concepts, including participatory design principles, the Consolidated Framework for Implementation Research (CFIR), and the Nonadoption, Abandonment, Scale-up, Spread, and Sustainability (NASSS) framework (5, 7).

For example, one concrete analytical pathway involved the neonatal risk-prediction project. The study was implemented across three health facilities and included about 394 newborn assessments over four months. Although usability findings were positive, only 44% of respondents reported changes in referral practices, while staffing shortages, high workload, and reliance on hybrid paper–digital systems constrained integration into routine care (21, 42). These findings were initially coded as workflow integration, workload, and infrastructure constraints. Comparison with findings from the Bright Health project, which identified unreliable electricity and connectivity, maintenance capacity, and governance as important implementation considerations (43, 44), supported a recurring cross-project pattern: technical feasibility and user acceptance were insufficient for implementation without corresponding workflow, organizational, and system readiness. This pattern was interpreted in relation to HCD and implementation concepts and subsequently mapped to the Meso and Macro levels, particularly the Evaluate, Implement, and Scale phases of the proposed framework. This example illustrates how project-level empirical evidence was retained as the basis for cross-project comparison and how the resulting methodological pattern was translated into a specific level and phase of the framework.

During the first phase, each study was examined independently to identify methodological processes and implementation experiences relevant to digital health development and deployment. Particular attention was given to contextual inquiry, stakeholder engagement, participatory co-design activities, usability and user experience evaluation, workflow integration, implementation readiness, governance, and sustainability. Findings from each study were subjected to open coding, allowing methodological concepts and implementation characteristics to be identified while preserving the contextual integrity of individual projects (51).

The second phase involved cross-project methodological synthesis using a constant comparative approach. The analysis systematically compared codes and preliminary themes identified within individual studies to identify recurring methodological processes, similarities, contextual differences, and explanatory relationships across diverse implementation settings. Rather than synthesizing intervention outcomes, the analysis focused on identifying methodological processes that consistently supported successful digital health implementation across community, clinical, and health system contexts. Related concepts were iteratively grouped into higher-order analytical themes through repeated comparison and analytical discussion (39, 52).

During the final phase, the synthesized themes were translated into the proposed multi-level human-centered design framework through iterative conceptual mapping. Each higher-order methodological theme was traced back to the contributing project-level findings and then mapped according to (1) the socio-technical level at which the methodological issue primarily operated, i.e., user/micro, workflow/meso, or system/macro, and (2) the implementation lifecycle phase in which it was most directly relevant. Themes that spanned more than one level or phase were retained as cross-level or iterative relationships rather than forced into a single category. For example, findings on usability and stakeholder experience informed the micro/user level, while findings on workflow integration and organizational readiness informed the meso/workflow level; infrastructure, governance, and sustainability findings informed the macro/system level. This iterative mapping process provided an explicit analytical pathway from empirical observations to the corresponding framework components and their interrelationships.

Relationships, dependencies, and iterative feedback mechanisms among these levels were examined repeatedly to ensure conceptual coherence, explanatory consistency, and faithful representation of the empirical evidence. Framework refinement continued through iterative comparison and conceptual review until the authors judged that the higher-order concepts provided a coherent representation of the recurring methodological patterns that were identified and interpreted by the research team through iterative engagement with the empirical corpus (53). Figure 2 illustrates the methodological process for deriving the proposed framework from the empirical corpus.

**Figure 2:**
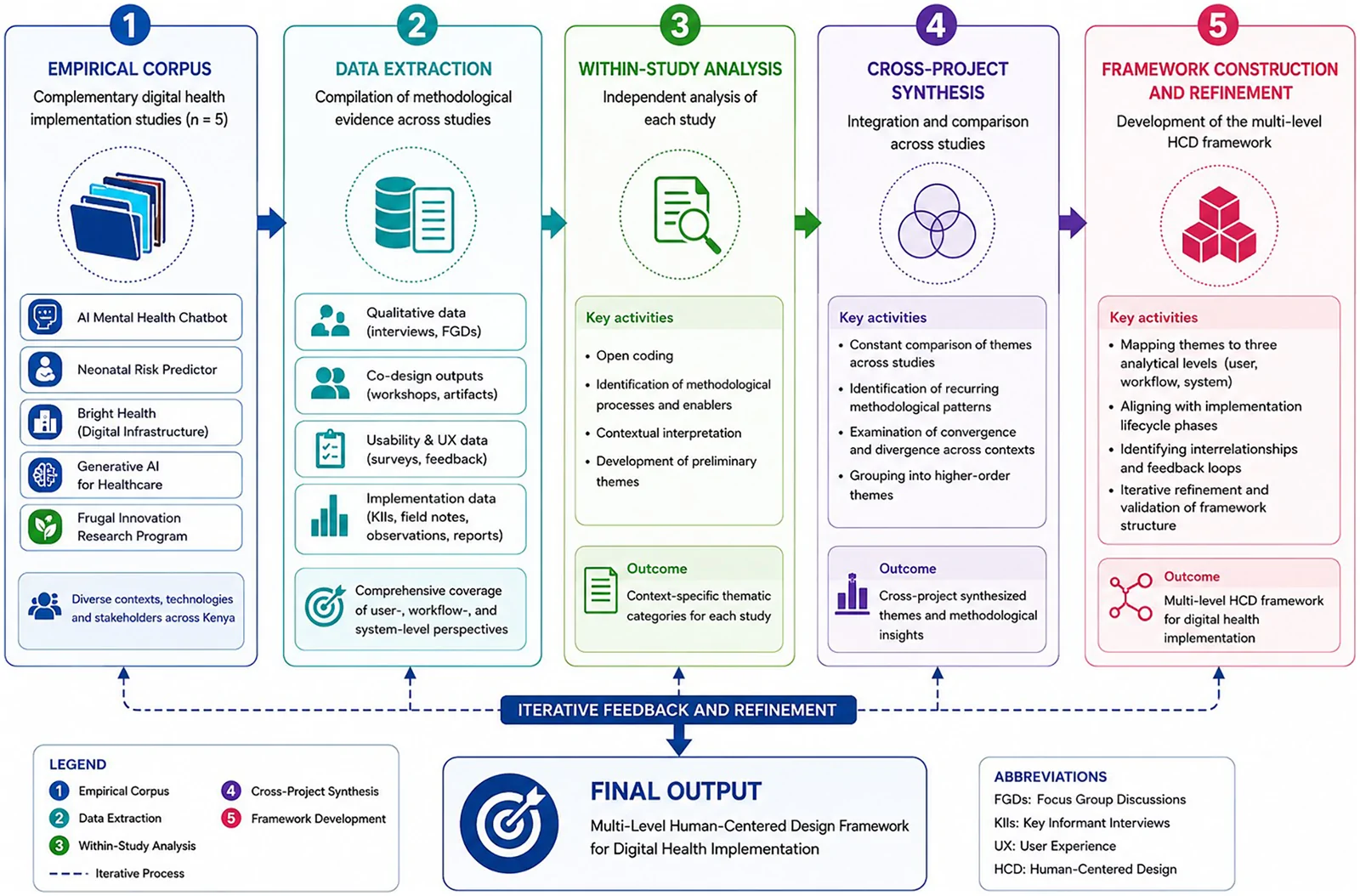
Cross-project methodological synthesis process used to develop the proposed multi-level human-centered design framework.

Figure 2 illustrates the sequential methodological process underpinning framework development, beginning with the empirical corpus of digital health implementation studies, followed by within-study analysis, cross-project methodological synthesis, iterative framework construction, and conceptual refinement, culminating in the final multi-level framework.

### Analytical transparency

Framework development was documented through an iterative analytical audit trail. Methodological themes identified through the analysis were continuously compared across studies and refined through repeated examination of the empirical findings and relevant implementation science literature. Rather than conducting *de novo* qualitative coding, the synthesis integrated previously analyzed findings from the included studies using constant comparison and abductive reasoning. Analytical decisions were documented throughout framework development to enhance transparency and methodological consistency. Because the purpose of the study was methodological framework development rather than qualitative meta-synthesis, formal inter-rater reliability assessment and independent recoding were not undertaken. Because the objective was methodological framework development rather than primary qualitative analysis, methodological triangulation, constant comparison across studies, and iterative framework refinement were used to enhance analytical credibility instead of independent recoding.

### Reflexivity and researcher positioning

Because all studies included in the methodological synthesis were conducted within the same research program, the framework development considered reflexivity throughout. The lead researcher was directly involved in conceptualizing, designing, implementing, and analyzing the included studies, providing deep contextual understanding of the research settings, stakeholder perspectives, and implementation processes. Accordingly, the identification of participatory and human-centered design as recurring methodological patterns should be interpreted in light of the team's established methodological orientation rather than assumed to represent the only possible cross-project pattern. While this facilitated in-depth interpretation of methodological processes across studies, it also introduced the potential for interpretive bias (54).

To strengthen analytical credibility, I integrated reflexive practice throughout the synthesis. Interpretations were continuously compared against original study findings, methodological documentation, and implementation evidence to ensure that higher-order themes remained grounded in the empirical corpus. Iterative comparison across studies, transparent documentation of analytical decisions, and repeated refinement of conceptual categories contributed to the credibility and coherence of the resulting framework. This reflexive approach sought to balance contextual familiarity with methodological rigor while maintaining transparency throughout framework development (55).

### Rigor and trustworthiness

Several strategies enhanced the rigor and trustworthiness of the methodological synthesis. Methodological triangulation was achieved by integrating complementary qualitative, quantitative, usability, implementation, and project documentation from the included studies (56). This diversity of evidence enabled examination of methodological processes from multiple perspectives and strengthened the framework's credibility.

Credibility was further supported through constant comparison across studies, in which themes were repeatedly examined against the original empirical evidence to ensure that conceptual interpretations accurately reflected the methodological experiences represented in the research corpus (57). Analytical decisions were documented throughout the synthesis, providing an audit trail that enhanced the transparency and reliability of the framework's development.

Theoretical triangulation was achieved by interpreting findings alongside established implementation science frameworks, including CFIR and NASSS, while simultaneously drawing upon principles of human-centered design and participatory research. This iterative interaction between empirical evidence and existing theory strengthened the explanatory power of the proposed framework without constraining the development of inductive themes (46).

Finally, synthesizing evidence across diverse digital health implementation contexts, including community-based, clinical, infrastructural, and artificial intelligence applications, supported transferability. Although all studies were undertaken within Kenya, the diversity of technologies, stakeholder groups, and implementation settings increases the potential applicability of the methodological principles to comparable digital health contexts in other resource-constrained health systems.

## Results

### Overview of the cross-project methodological synthesis

The cross-project methodological synthesis integrated empirical evidence generated from a coherent corpus of complementary digital health implementation studies conducted across multiple healthcare contexts in Kenya. Collectively, these studies covered diverse digital health interventions, including adolescent mental health, neonatal clinical decision support, digital health infrastructure, generative artificial intelligence, and frugal digital health innovation. Despite differences in technological focus, implementation settings, and stakeholder groups, all studies shared common methodological principles grounded in human-centered design, user-centered design, participatory research, implementation science, or design science approaches.

Cross-project comparison demonstrated substantial methodological convergence despite variation in intervention objectives and implementation contexts. Across the included studies, recurring methodological processes consistently emphasized contextual understanding, stakeholder engagement, participatory co-design, iterative refinement, usability evaluation, workflow integration, organizational readiness, governance, and long-term sustainability. These methodological characteristics appeared across community-based, clinical, and health system implementation settings, suggesting they represent transferable principles rather than project-specific activities.

Rather than synthesizing intervention outcomes, the analysis focused on identifying recurring methodological processes that consistently supported successful digital health implementation. Through iterative comparison across studies, the analysis progressively consolidated individual methodological activities into higher-order themes representing common implementation processes. These synthesized themes subsequently formed the empirical foundation for developing the proposed multi-level human-centered design framework.

Comparing these methodological characteristics revealed recurring implementation processes that transcended individual technologies and implementation settings. These higher-order methodological themes are presented in the following section.

### Recurring methodological themes across projects

Cross-project comparison identified recurring methodological themes that consistently supported the design, implementation, and sustainability of digital health interventions across diverse contexts. Although individual studies addressed different technologies, healthcare settings, and stakeholder groups, the implementation lifecycle showed remarkably similar methodological processes. These recurring processes extended beyond traditional human-centered design activities and reflected broader interactions between users, organizational workflows, and health systems.

The first recurring theme was contextual understanding, which consistently underpinned digital health implementation. Across all included projects, early engagement with end users, healthcare professionals, community members, and other stakeholders helped identify contextual needs, implementation barriers, workflow constraints, and sociocultural factors that influence technology adoption. This finding reinforced the importance of understanding implementation environments before commencing technology development or deployment and highlighted contextual inquiry as a prerequisite for successful human-centered design.

The second recurring theme involved participatory stakeholder engagement through co-design and iterative refinement. Across settings, meaningful involvement of intended users throughout the design process improved usability, acceptability, contextual relevance, and stakeholder ownership. Rather than functioning as isolated design activities, co-design workshops, usability testing, and iterative feedback cycles formed continuous learning processes that progressively refined digital health interventions throughout development and implementation.

Workflow integration was the key methodological bridge between user-centered design and successful implementation. Across community- and facility-based studies, implementation success depended not only on user acceptance but also on alignment with existing clinical workflows, organizational processes, and care delivery patterns. Interventions that accommodated routine practices, minimized cognitive burden, and integrated naturally into service delivery were consistently more likely to demonstrate implementation readiness and long-term sustainability.

The synthesis further demonstrated that organizational readiness and system-level governance were essential determinants of successful implementation. Factors including leadership support, workforce capacity, digital infrastructure, policy alignment, financing, and governance mechanisms consistently influenced digital health interventions' ability to transition from pilot implementation to sustainable integration and scale-up. These findings emphasized that implementation success depends upon interactions between technological innovation and the broader health system in which implementation occurs.

Collectively, these recurring methodological themes showed that successful digital health implementation requires coordinated attention to user experiences, workflow processes, and system readiness throughout the implementation lifecycle. Rather than representing independent methodological components, these themes interacted dynamically across projects, providing an empirical basis for developing a multi-level implementation framework that integrates human-centered design with implementation science principles. Table 4 summarizes the recurring methodological themes identified through the cross-project synthesis and their contributions to framework development.

**Table 4:** Recurring methodological themes identified through the cross-project synthesis and their contributions to framework development.

Recurring methodological theme	Evidence across projects	Contribution to framework development
Contextual understanding	Stakeholder interviews, focus groups, contextual inquiry, needs assessments	Established the Understand phase and reinforced the importance of contextual exploration before intervention design.
Participatory co-design	Co-design workshops, iterative stakeholder engagement, prototype refinement	Informed the Co-design and Prototype phases while emphasizing continuous stakeholder involvement.
Usability and iterative evaluation	Usability testing, user experience assessments, iterative feedback	Shaped the Evaluate phase and reinforced continuous refinement throughout implementation.
Workflow integration	Workflow mapping, implementation assessments, task alignment	Established the Workflow (Meso) analytical level linking user-centered design with implementation.
Organizational readiness	Capacity assessment, implementation planning, infrastructure evaluation	Contributed to the System (Macro) level by emphasizing implementation readiness.
Governance and sustainability	Policy alignment, leadership engagement, financing, scale-up planning	Informed the Implement and Scale phases and strengthened long-term sustainability considerations.

Integrating these recurring methodological themes enabled the development of the proposed multi-level human-centered design framework, described in the following section.

### Development of the multi-level human-centered design framework

The cross-project synthesis identified recurring methodological themes and integrated them into a unified, multi-level human-centered design framework for digital health implementation. Framework development followed the principle that successful implementation depends not on user-centered design alone, but on continuous alignment among users, organizational workflows, and health systems throughout the implementation lifecycle. Consequently, the framework extends conventional human-centered design by explicitly incorporating workflow integration and system readiness as essential components of implementation success.

The framework is organized around six sequential yet iterative implementation phases: Understand, Co-design, Prototype, Evaluate, Implement, and Scale. These phases represent a continuous implementation lifecycle rather than a strictly linear process, recognizing that digital health interventions require ongoing refinement in response to user feedback, contextual changes, organizational learning, and evolving implementation environments. Iterative feedback mechanisms operate throughout the lifecycle, enabling continuous adaptation and improvement.

Across each implementation phase, the framework incorporates three interconnected analytical levels representing users (micro), workflows (meso), and health systems (macro). The user level emphasizes understanding stakeholder needs, experiences, usability, accessibility, and engagement. The workflow level focuses on integration into routine practice through task alignment, cognitive workload, interoperability, communication, and organizational processes. The system level addresses broader implementation determinants including governance, leadership, digital infrastructure, workforce capacity, financing, policy, and long-term sustainability. These levels do not function independently; they interact continuously throughout implementation, with changes at one level influencing implementation processes at the others.

The resulting framework conceptualizes digital health implementation as a dynamic sociotechnical process in which successful adoption depends on continuous interaction among users, workflows, and health systems across the full implementation lifecycle. By integrating principles from human-centered design and implementation science within a single methodological structure, the framework provides practical guidance for designing, implementing, evaluating, and scaling digital health interventions in complex healthcare environments. Figure 3 presents the proposed multi-level human-centered design framework derived from the cross-project methodological synthesis.

**Figure 3:**
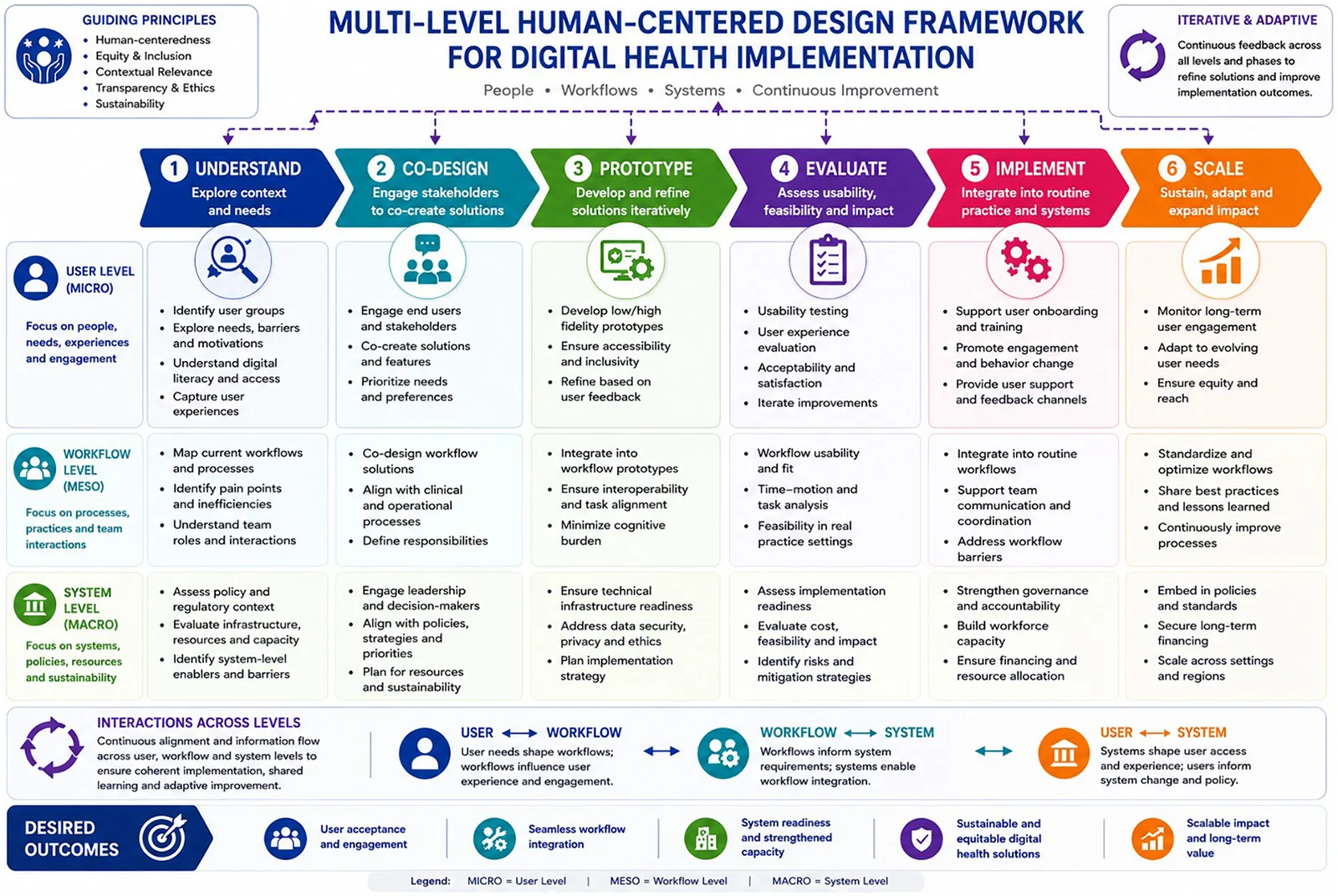
Proposed multi-level human-centered design framework for digital health implementation derived from cross-project methodological synthesis.

Figure 3 illustrates the proposed framework, comprising six iterative implementation phases, Understand, Co-design, Prototype, Evaluate, Implement, and Scale, operating across three interconnected analytical levels: user (micro), workflow (meso), and system (macro). Continuous feedback mechanisms facilitate iterative refinement throughout the implementation lifecycle, supporting sustainable digital health implementation and scale-up.

### Illustrative application of the framework

The neonatal risk-prediction project provides a concrete example of how the proposed framework can be applied to an empirical implementation context (21, 42). The project was implemented across three health facilities and included approximately 394 newborn assessments over four months. Despite positive usability findings, the neonatal risk-prediction tool's translation into routine referral practices remained limited. The findings highlighted a gap between user acceptance and practical integration, shaped by staffing constraints, workload pressures, and continued reliance on hybrid paper–digital systems (21, 42). These findings illustrate why usability alone is insufficient to determine implementation readiness. At the micro/user level, the intervention must be understandable and usable by healthcare professionals; at the meso/workflow level, referral practices, workload, documentation, and integration with existing routines must be considered; and at the macro/system level, infrastructure, organizational readiness, governance, and sustainability must be assessed. The example therefore demonstrates how evidence from an implementation context can be examined across interconnected levels rather than interpreted solely in terms of usability or technical feasibility.

### Operational application of the framework

The framework is intended to guide implementation as an iterative, rather than strictly linear, process. Practitioners can begin by using the Understand phase to characterize users, workflows, organizational conditions, and system constraints through contextual inquiry, stakeholder interviews, workflow observation, document review, and other appropriate methods. In the Co-design phase, teams translate findings into priorities and requirements through participatory workshops, co-design activities, journey mapping, and other stakeholder-engagement methods. The Prototype phase then uses these requirements to develop and iteratively refine the intervention, with rapid feedback from intended users and other relevant stakeholders. The Evaluate phase examines usability, acceptability, workflow fit, implementation readiness, and other context-appropriate outcomes using methods such as usability testing, user experience assessment, workflow evaluation, and implementation assessment. The Implement phase focuses on integration into routine practice, including workflow alignment, training, organizational readiness, governance, and infrastructure requirements. Finally, the Scale phase examines sustainability, institutionalization, adaptation, resource requirements, and the conditions needed to expand to additional settings.

Across all six phases, practitioners should examine the three levels concurrently. At the micro level, assessment may include usability, accessibility, acceptability, engagement, trust, and user experience. At the meso level, assessment may include workflow fit, task alignment, cognitive workload, interoperability, coordination, and disruption to routine practice. At the macro level, assessment may include organizational readiness, governance, infrastructure, workforce capacity, financing, policy alignment, sustainability, and scalability. Progress should not be interpreted as advancement through phases alone; rather, implementation readiness is indicated by increasing alignment across the three levels. Persistent misalignment at any level should trigger iterative reassessment and, where necessary, a return to an earlier phase.

Table 5 provides an operational guide linking each framework phase to representative activities, methods, and indicators across the three levels. The specific methods and indicators are not mandatory; adapt them to the intervention, implementation context, available resources, and intended users.

**Table 5:** Operational application of the multi-level human-centered design framework.

Framework phase	Core practitioner question	Representative activities and methods	Micro: user-level assessment	Meso: workflow-level assessment	Macro: system-level assessment
Understand	What needs, practices, and contextual conditions shape the intervention?	Contextual inquiry, interviews, observation, stakeholder mapping, document review	Needs, experiences, accessibility, trust	Tasks, workflow dependencies, workload	Readiness, infrastructure, governance, resources
Co-design	What should be designed or adapted with stakeholders?	Co-design workshops, participatory design, journey mapping, requirements elicitation	User priorities and preferences	Workflow requirements and task alignment	Organizational and system requirements
Prototype	Does the proposed solution fit identified needs and contexts?	Prototyping, iterative refinement, scenario testing, usability walkthroughs	Usability, acceptability, user experience	Workflow fit and disruption	Technical feasibility and organizational compatibility
Evaluate	Does the intervention work for users and within the intended context?	Usability testing, UX evaluation, workflow assessment, implementation assessment	Usability, engagement, satisfaction	Workflow performance, coordination, interoperability	Readiness, governance, infrastructure, implementation conditions
Implement	Can the intervention be integrated into routine practice?	Implementation planning, training, workflow integration, stakeholder feedback, adaptation	Adoption and continued engagement	Integration, workload, task coordination	Organizational readiness, governance, workforce, infrastructure
Scale	Can the intervention be sustained, adapted, and expanded?	Sustainability assessment, adaptation planning, scale-up assessment, monitoring	Continued acceptability and engagement	Transferability and workflow adaptation	Sustainability, financing, policy alignment, institutionalization, scale-up

### Cross-project methodological insights

Beyond developing the proposed framework, the cross-project synthesis generated several methodological insights relevant to designing and implementing digital health interventions. Despite substantial differences in technologies, target populations, and implementation settings, the included studies consistently showed that successful digital health implementation depends on coordinated alignment among user needs, organizational workflows, and broader health system capacity, rather than optimizing any single component in isolation.

The synthesis further showed that human-centered design should be viewed as an ongoing implementation strategy rather than a discrete design activity. Although participatory approaches such as contextual inquiry, co-design, usability testing, and iterative refinement remained fundamental throughout the included projects, their effectiveness depended on simultaneously considering workflow integration, organizational readiness, governance structures, and implementation planning. Consequently, human-centered design served not only as a design methodology but also as a mechanism to facilitate implementation, organizational learning, and long-term sustainability.

Differences across implementation contexts highlighted the framework's adaptability. Community-based interventions primarily emphasized stakeholder engagement, contextual understanding, trust, accessibility, and sustained user participation, whereas clinical implementations required greater attention to workflow integration, interoperability, cognitive workload, governance, and institutional readiness. Nevertheless, these contextual differences reflected variations in emphasis rather than differences in the underlying methodological principles, supporting the framework's applicability across diverse digital health settings.

Collectively, these findings indicate that effective digital health implementation requires a lifecycle-oriented methodological approach that integrates participatory design, implementation science, and systems thinking within a unified framework. Rather than treating design, implementation, evaluation, and scale-up as independent phases, the proposed framework conceptualizes them as interconnected processes supported by continuous feedback, adaptation, and organizational learning across user, workflow, and system levels. To summarize the principal methodological insights from the cross-project synthesis, Table 6 presents recurring implementation lessons and their implications for framework development.

**Table 6:** Cross-project methodological insights and their implications for the proposed multi-level human-centered design framework.

Cross-project insight	Evidence across projects	Implication for framework development
Context precedes technology	All projects began with contextual inquiry and stakeholder engagement	Reinforced the **Understand** phase as the foundation of implementation.
Co-design is continuous	Iterative engagement occurred throughout development and implementation	Positioned co-design as a continuous process rather than a single activity.
Workflow determines implementation success	Clinical and community projects highlighted the importance of workflow alignment	Established the **Workflow (Meso)** level as the bridge between users and systems.
System readiness influences sustainability	Governance, infrastructure, workforce capacity, and financing consistently affected implementation	Strengthened the **System (Macro)** level and the **Implement** and **Scale** phases.
Feedback drives continuous improvement	All projects incorporated iterative refinement based on stakeholder input	Supported the framework's continuous feedback loop across all implementation phases.
Human-centered design extends beyond usability	Successful projects integrated participatory design with implementation planning and governance	Demonstrated the need for a lifecycle-oriented, multi-level methodological approach.

These cross-project methodological insights provide a basis for discussing the proposed framework's broader theoretical and practical contributions in relation to existing human-centered design and implementation science literature.

## Discussion

### Principal findings and methodological contribution

This study developed a multi-level human-centered design (HCD) framework for digital health implementation through a cross-project methodological synthesis of complementary studies conducted in Kenya. By integrating empirical evidence across diverse implementation contexts, the proposed framework extends conventional HCD beyond user-centered design to explicitly incorporate workflow integration and system readiness throughout the digital health implementation lifecycle. Rather than conceptualizing implementation as a sequence of isolated design activities, the framework presents digital health implementation as a dynamic, iterative sociotechnical process requiring continuous alignment among users, organizational workflows, and health systems.

A key methodological contribution of this study is demonstrating how evidence from multiple complementary implementation studies can be systematically synthesized to develop an implementation-oriented framework. While previous research has widely advocated HCD as an effective approach for improving usability, acceptability, and user engagement, comparatively limited attention has been given to methodological strategies for integrating HCD with implementation science across the complete implementation lifecycle (10, 58). The proposed framework addresses this gap by providing an empirically derived methodological structure linking participatory design, implementation readiness, governance, and scale-up within a unified conceptual model.

Importantly, the framework emphasizes that user-centered design alone cannot achieve successful implementation. Across the synthesized studies, implementation success consistently depended upon interactions among contextual understanding, stakeholder engagement, workflow integration, organizational readiness, governance, and continuous adaptation. These findings reinforce the view that digital health interventions should be conceptualized as complex sociotechnical systems rather than stand-alone technological products (6, 28, 29, 59). To highlight the proposed framework's principal methodological contributions relative to existing approaches, Table 7 summarizes its key innovations and anticipated implications for digital health implementation research and practice.

**Table 7:** Principal methodological contributions of the proposed multi-level human-centered design framework.

Methodological contribution	Advancement beyond existing approaches	Implications for digital health implementation
Lifecycle-oriented framework	Extends HCD from primarily design-oriented activities toward a continuous pathway spanning contextual understanding, co-design, prototyping, evaluation, implementation, and scale-up.	Supports continuity between design, implementation, evaluation, and scale-up.
Multi-level integration	Connects user (micro), workflow (meso), and system (macro) considerations throughout the lifecycle rather than treating these as separate analytical domains.	Encourages coordinated attention to users, work processes, organizations, and health-system conditions.
Integration of HCD and implementation science	Combines participatory design and iterative user engagement with implementation determinants and system readiness, complementing approaches such as CFIR and NASSS.	Bridges design activities with implementation planning and adaptation.
Sociotechnical workflow integration	Builds on the sociotechnical perspective of SEIPS while emphasizing participatory design and implementation-oriented lifecycle progression.	Links user experience and workflow integration with organizational and system readiness.
Cross-project methodological synthesis	Derives the framework from methodological evidence across multiple complementary digital health projects rather than from a single theoretical model or implementation evaluation.	Provides an empirically informed pathway for connecting methodological processes to framework components.
Continuous feedback and adaptation	Positions design and implementation as interconnected and iterative processes, allowing learning at one level or phase to inform adaptation at others.	Supports context-responsive implementation and longer-term sustainability.

The following section discusses the broader relationship between the proposed framework and existing human-centered design and implementation science frameworks.

### Relationship to existing human-centered design and implementation science frameworks

The proposed framework complements rather than replaces existing human-centered design and implementation science approaches. Its contribution lies primarily in connecting these perspectives across the implementation lifecycle and interacting system levels. Traditional HCD approaches emphasize understanding users, participatory co-design, iterative development, and usability, but offer less explicit methodological guidance on organizational readiness, governance, implementation, and scale-up. By contrast, implementation science frameworks such as CFIR provide structured approaches for examining implementation determinants and processes, while NASSS emphasizes the complexity and dynamic interactions influencing technology adoption, abandonment, scale-up, spread, and sustainability (6, 8, 17). SEIPS provides a strong sociotechnical and human factors perspective on work systems, processes, and patient journeys (22, 60).

The proposed framework differs in its primary purpose and organization. Rather than introducing another determinant-based framework or replacing these established approaches, it offers a lifecycle-oriented methodological pathway that links participatory HCD activities to implementation processes across three interacting levels: user (micro), workflow (meso), and system (macro). The six iterative phases, Understand, Co-design, Prototype, Evaluate, Implement, and Scale, are intended to serve as a practical methodological sequence that continuously connects and adapts insights from users, workflows, and system contexts.

The framework also positions HCD and implementation science as interconnected rather than sequential processes. This distinction matters because implementation challenges can require changes to the intervention, workflow, organizational arrangements, or system conditions, while changes at these levels can, in turn, generate new design requirements (6, 59). The framework extends HCD's methodological role beyond early-stage design and usability to continuous implementation learning, while retaining the contextual and sociotechnical strengths of implementation science and human factors approaches.

Accordingly, the framework's novelty is not that it introduces user, organizational, workflow, or system considerations independently; these are well established across HCD, CFIR, NASSS, and SEIPS. Rather, its contribution is the explicit integration of these considerations into a single, empirically derived, lifecycle-oriented methodological pathway for designing, evaluating, implementing, and scaling digital health interventions (17, 18, 60). The cross-project synthesis also provides an empirical basis for linking recurring methodological processes across implementation contexts. The framework should be understood as a complementary integration and operationalization of established perspectives, not a replacement.

Building on these methodological advances, the following section discusses the practical implications of the proposed framework for research, policy, and digital health implementation.

### Implications for research, practice, and policy

The proposed framework has several important implications for digital health research. First, it provides researchers with a structured methodological approach that integrates human-centered design and implementation science throughout the entire implementation lifecycle, rather than treating them as separate methodological traditions. This integrated perspective may facilitate more comprehensive evaluation of digital health interventions by encouraging simultaneous consideration of user experience, workflow integration, organizational readiness, and system-level implementation determinants. Consequently, future digital health studies may benefit from adopting lifecycle-oriented methodological approaches that extend beyond usability assessment and pilot implementation to include sustainability, governance, and scale-up from the outset (5, 6). In practice, teams can use the framework as an implementation planning and reflection tool: review each lifecycle phase against the micro, meso, and macro dimensions, document evidence of alignment or unresolved barriers, and use identified gaps to guide iterative adaptation before progressing or returning to an earlier phase.

The framework also has practical implications for digital health developers, implementation teams, clinicians, and healthcare organizations. By explicitly incorporating user, workflow, and system perspectives throughout implementation, the framework encourages multidisciplinary collaboration among designers, healthcare professionals, implementation specialists, policymakers, and end users. Such collaboration may reduce implementation failures stemming from poor workflow integration, insufficient stakeholder engagement, or limited organizational readiness, thereby increasing the likelihood of sustainable adoption in routine healthcare practice.

From a policy perspective, the framework highlights governance, leadership, workforce capacity, digital infrastructure, financing, and implementation planning as integral components of digital health innovation, not activities that occur only after technology development. Policymakers and health system leaders may use the framework to support strategic planning, implementation readiness assessments, resource allocation, and national digital health programs, particularly within resource-constrained health systems where efficient implementation and long-term sustainability are essential (4).

Another implication of the proposed framework concerns the ethical governance of participatory digital health implementation. Human-centered design should extend beyond stakeholder engagement to ensure that participation is meaningful, equitable, and ethically conducted throughout the implementation lifecycle, particularly when interventions involve vulnerable populations such as adolescents, neonates, and other underserved communities. Researchers and implementation teams should actively consider issues of informed consent, power asymmetries between researchers and participants, data privacy, confidentiality, and responsible data governance when designing, implementing, and evaluating digital health interventions (61–63). Embedding these ethical considerations across the user, workflow, and system levels can strengthen trust, promote transparency, reduce tokenistic participation, and support more equitable and sustainable digital health implementation.

Although the framework was developed through empirical evidence generated in Kenya, the methodological principles underpinning it are likely applicable across a broad range of digital health contexts. Future research should evaluate the framework prospectively across countries, healthcare systems, technologies, and implementation settings to assess its generalizability, adaptability, and contribution to implementation outcomes. Such evaluations will further strengthen the evidence base supporting lifecycle-oriented human-centered implementation methodologies. Table 8 summarizes the proposed framework's potential applications for key stakeholder groups.

**Table 8:** Potential applications of the proposed multi-level human-centered design framework across stakeholder groups.

Stakeholder group	Potential application of the framework
Researchers	Design and evaluate digital health interventions using an integrated lifecycle methodology.
Digital health developers	Incorporate user, workflow, and system considerations throughout technology development.
Healthcare professionals	Facilitate workflow integration and improve implementation within routine clinical practice.
Health service managers	Assess organizational readiness, infrastructure, governance, and implementation capacity.
Policymakers	Inform strategic planning, digital health governance, implementation readiness, and scale-up policies.
Funding agencies	Evaluate implementation readiness and long-term sustainability when prioritizing investments in digital health innovation.

While the proposed framework offers several methodological and practical advantages, interpret it alongside the strengths and limitations of the present study.

### Strengths and limitations

This study has several important strengths. First, the proposed framework was developed through a structured cross-project methodological synthesis of complementary digital health implementation studies rather than from a single intervention or purely theoretical model. This approach identified recurring methodological principles across diverse technologies, stakeholder groups, and implementation settings, strengthening the framework's empirical foundation and methodological credibility. Second, the synthesis integrated perspectives from human-centered design, implementation science, participatory research, and design science, providing a comprehensive methodological approach that bridges disciplines frequently considered separately in the digital health literature (65, 66). Third, the framework drew on empirical evidence from community-based, clinical, and health-system implementation contexts, enhancing its relevance to complex real-world digital health environments.

Several limitations should be considered when interpreting this framework. First, the empirical corpus was purposively drawn from five digital health projects conducted by the same research team in Kenya. The framework reflects the methodological experiences, implementation contexts, and design practices represented in these projects and may not capture alternative approaches or priorities that would emerge from projects conducted by other research teams or in other settings. Although the projects covered diverse health domains, technologies, stakeholder groups, and implementation contexts, transferability beyond the Kenyan and resource-constrained settings represented here should be considered cautiously and assessed through application in other contexts.

Second, researcher involvement across the source projects introduces potential analytic and interpretive bias. The cross-project synthesis was conducted retrospectively using previously generated and analyzed project findings, rather than through an independent secondary analysis by researchers external to the source projects. Reflexive consideration of researcher positioning, constant comparison across projects, methodological triangulation, and documentation of analytical decisions strengthened credibility; however, these procedures cannot eliminate the possibility that the research team's methodological preferences influenced the patterns identified and their translation into framework components.

Third, the synthesis was methodological rather than a prospective validation study. The framework has not yet been independently tested across new implementation settings or evaluated longitudinally through full-scale implementation. Its utility, completeness, and transferability require prospective assessment. Finally, generative artificial intelligence was used transparently and in a limited supportive capacity to support language editing, manuscript organization, and the generation of illustrative figures. AI tools did not generate empirical findings, conduct the cross-project synthesis, determine analytical themes, or make substantive methodological decisions. The authors remained responsible for all substantive interpretation, framework development, and final manuscript decisions.

Fourth, the same research team conducted the five projects, creating a potential source of selection and interpretive bias (21, 37, 38, 41–45, 67–70). The team's established use of human-centered and participatory approaches may have contributed to the prominence of these methodological patterns in the synthesis, and a different research team or methodological tradition might identify different cross-project themes.

## Conclusion

This study developed a multi-level human-centered design framework for digital health implementation through a cross-project methodological synthesis of complementary digital health implementation studies. By integrating empirical evidence across multiple implementation contexts, the framework extends conventional human-centered design beyond participatory design and usability to explicitly incorporate workflow integration, organizational readiness, governance, and scalability throughout the implementation lifecycle.

The proposed framework contributes methodologically by showing how human-centered design and implementation science can be systematically integrated into a unified, lifecycle-oriented approach to digital health implementation. Rather than viewing design, implementation, evaluation, and scale-up as independent activities, the framework conceptualizes them as interconnected, iterative processes that require continuous alignment among users, organizational workflows, and health systems. This integrated perspective provides a practical methodological foundation for designing, implementing, evaluating, and sustaining digital health interventions within complex healthcare environments.

Although the framework was developed using empirical evidence from Kenya, its underlying methodological principles are intended to be transferable across diverse digital health settings. Future research should prospectively validate and refine the framework across diverse technologies, healthcare systems, and geographical contexts, while exploring its contributions to implementation effectiveness, sustainability, and equitable digital health transformation.

As digital health evolves from isolated technological innovations toward integrated health system transformation, implementation methodologies must similarly evolve beyond user-centered design alone. The proposed framework provides a structured methodological pathway that bridges human-centered design and implementation science, offering researchers, practitioners, developers, and policymakers a practical approach for achieving sustainable, scalable, and context-responsive digital health implementation.

## Data Availability

The original contributions presented in the study are included in the article/Supplementary Material, further inquiries can be directed to the corresponding author.
